# Fostering internal assets to mitigate suicidal behaviors among young black sexual minority males aged 14–24

**DOI:** 10.1186/s12889-025-24131-y

**Published:** 2025-08-20

**Authors:** Donte T. Boyd, Sally Kirklewski, Juan L. Benavides, Camille R. Quinn, Ashima Saigal, Samantha V. Hill

**Affiliations:** 1 College of Social Work, The Ohio State University, 1047 College RD, #325K, Columbus, OH 43215 USA; 2 Department of Social Behavioral Sciences, School of Public Health, Yale University, New Haven, CT USA; 3 School of Social Work, University of Michigan, Ann Arbor, MI USA; 4 School of Medicine, Emory University, Atlanta, GA USA

**Keywords:** Internal assets, Suicidal behaviors, Adolescents, Young Adults, Black sexual minority males

## Abstract

**Objective:**

Suicidal behaviors among young Black sexual gender minority males ages 14–24 is a pressing issue that requires urgent attention. This study aims to fill a significant gap in the literature by investigating how internal assets impact suicidal behaviors among young Black sexual minority men (SMM) ages 14–24.

**Methods:**

This study utilized a cross-sectional survey of young Black SMM (*N* = 538) from December 1, 2023, to January 31, 2024, to examine the influence of internal assets (such as positive values, positive identity) on suicidal behaviors. A logistic regression was performed to examine the associations between internal assets and suicidal behaviors.

**Results:**

Our findings indicated that positive identity was associated with lower levels of suicidal behaviors (β = − 0.29; *p* < 0.001). Positive values (β = − 0.24; *p* < 0.001) and social competencies (β = − 0.29; *p* < 0.001) were also associated with fewer suicidal behaviors.

**Conclusions:**

Our results indicate that internal assets play a significant role in influencing suicidal behaviors among young Black sexual gender minority males. By promoting positive values, positive identity, and social competencies, we can help build resilience and reduce the risk of suicide among Black SMM.

## Background

Suicide persists as a significant public health concern and remains the second leading cause of death among youth ages 10–24, claiming more young lives than all childhood medical disorders combined [1–5]. Particularly alarming are the statistics among Black LGBTQ youth, 44% of whom have seriously considered suicide in the past 12 months. This rate increases to 59% among Black transgender and nonbinary youth [6]. Furthermore, studies have shown that 27% of Black SMM ages 18–29 have attempted suicide, and 33% have made plans to die by suicide [7–9]. In the United States, there is growing concern for the well-being of LGBTQ youth, including Black sexual minority men, as more than 1.8 million individuals ages 13–24 seriously contemplate suicide each year [10, 11]. This highlights the critical need for dedicated research on young Black SMM ages 14–24.

Suicide rates among Black SMM ages 14–24 represents a critical area of concern that underscores a significant public health issue. Black SMM within this age group encounter intersectional challenges, including navigating sexual identity formation in potentially unsupportive environments and dealing with societal stigmas that can compound mental health risks [12, 13]. For Black SMM, these difficulties often are compounded by additional layers of racial discrimination and cultural pressures, making their situation uniquely challenging [14]. The intersection of racial and sexual minority status can lead to highly interrelated stressors, impacting their mental health, development, and well-being significantly. These experiences are not only isolating but also are recognized risk factors for suicidal ideation and behavior [12, 13]. Research also points to the importance of tailored interventions that address the unique cultural and social contexts of young Black SMM ages 14–24, emphasizing the need for more nuanced interventions and studies in this area [10, 11].

The lack of representation and visibility of young Black SMM in mainstream LGBTQ narratives and resources can lead to a sense of alienation and a lack of accessible support systems [15]. The data thus not only reflects the severity of the mental health crisis among LGBTQ youths but also highlights the disparities within this group, emphasizing the need for intersectional approaches in research and intervention design. In response to these challenges, the Developmental Assets Framework (DAF) emerges as a potential solution. DAF categorizes 40 developmental assets into two main categories: 20 external assets, which include community and family support, and 20 internal assets, which encompass positive identity and social competencies [15, 16]. The framework posits that more assets are inversely related to risky behaviors and positively associated with optimal youth outcomes.

Despite the comprehensive nature of the DAF, its specific application to Black SMM ages 14–24 remains insufficiently explored. This demographic is uniquely impacted by such factors as identity-based discrimination, financial insecurity, substance misuse, and social isolation, which collectively elevate suicide risk [17, 18]. Existing literature has identified external assets and their association with suicidal behaviors such as supportive familial and educational environments and a robust sense of community; however, these studies often do not focus specifically on the experiences of young Black SMM [7–9]. Recognizing this gap, this is the first paper to explore the application of internal assets possessed by young Black SMM ages 14–24, particularly examining how internal assets (academic engagement, positive values, positive identity, and social competencies) influence suicidal behaviors. This research aims to contribute to developing targeted strategies and interventions that can effectively support the mental health and well-being of young Black SMM.

### Theoretical framework

The Developmental Assets Framework (DAF), developed by the Search Institute in the 1990s, emerged in response to prevailing deficit-based models in adolescent research. DAF is a critical model in the field of positive youth development (PYD), emphasizing the interplay of external influences and internal strengths that foster adolescent well-being [10, 11]. Rather than focusing solely on risk behaviors, the DAF delineates 40 empirically supported assets—20 internal e.g., positive identity, future orientation, social competence) and 20 external (e.g., support, empowerment, constructive use of time)—that promote resilience, well-being, and healthy transitions to adulthood [10, 11]. These elements are essential in promoting safety, engagement, and a sense of belonging, helping to mitigate potential risk behaviors. Internal assets focus on individual qualities such as resilience, self-regulation, empathy, and a sense of purpose, which are vital for personal growth and positive life choices [10, 11]. This framework serves as a guiding tool for educators, community leaders, and policymakers, advocating for a systematic approach to youth development that enhances community support while cultivating personal capabilities. Research indicates that youth with a higher number of developmental assets are more likely to engage in positive behaviors and avoid risky situations [12, 13]. By highlighting the shared responsibility of families, schools, and communities in promoting these assets, the DAF fosters the resilience and well-being vital for the success of the next generation, thereby contributing to healthier and more engaged societies [11, 14].

Current research highlights external assets, including positive family support and parent communication, are associated with lower rates of suicidal behaviors among young Black SMM aged 18–29. These findings reinforce the critical role that support systems play in the development and well-being of young people. Conversely, internal assets encompass personal attributes, including a commitment to learning, self-efficacy, and positive values [15]. A study by Wiium et al. indicates that internal assets create a dynamic relationship between the individual and their supportive environment, facilitating adaptive developmental regulations that allow both youth and their contexts to thrive collectively [16]. Internal assets have been associated with improved resilience against anxiety and stress, as observed by Gómez-Baya et al., indicating their vital role in fostering mental health among youth [19]. Also, DAF has been broadly applied to community contexts with racially and socioeconomically diverse adolescents, but it has been limited in research explicitly addressing the well-being among Black SMM—a population often situated at the intersection of multiple marginalized identities and structural stressors. Overall, the presence of positive internal assets can potentially play a significant role in building resilience and fostering protective factors that affect how young Black SMM respond to stress and manage negative emotions. Further, few studies have utilized DAF to understand protective factors in the context of suicidal risk, particularly among youth navigating cumulative racial and cultural invalidations. By nurturing these internal assets, young Black SMM can create a supportive environment that significantly reduces the risk of suicidal behaviors among youth. The interconnected nature of internal assets highlights the importance of a holistic approach to promoting young Black SMM health and well-being.

### Current study

Young Black sexual minority males face significant mental health challenges, including disproportionately high rates of suicidal ideation and behavior. Identifying the assets that contribute to reduce risks is essential for developing effective strength based prevention and intervention strategies. Research has identified developmental assets—such as positive identity, academic engagement, positive values, and social competencies— as protective factors that can enhance positive mental health outcomes. This study aims to examine how these developmental assets may mitigate suicide risk among young Black SMM aged 14 to 24. To this end, we propose the following hypotheses to investigate the relationship between developmental assets and suicidal behaviors among young Black MSM aged 14–24:

- H_1_: Positive identity will be associated with lower suicidal behaviors.
- H_2_: Academic engagement will be associated with lower suicidal behaviors.
- H_3_: Positive values will be associated with lower suicidal behaviors.
- H_4_: Social competencies will be associated with lower suicidal behaviors.

## Methods

### Study procedures and recruitment

This study used data from a larger study that examined the effect of developmental assets on sexual, physical, and mental health and suicidal behaviors among young Black SMM ages 14–24 (N = 538) [17, 18, 20]. The survey was programmed with Qualtrics software. An anonymous link was generated and included on a recruitment flyer. The research team used a paid advertisement (flyer) that was distributed via social media platforms (Facebook and Twitter) and shared with community-based organizations and schools in three Midwestern cities. For community-based organizations, the research team shared the flyer with community health workers, who distributed the flyer to eligible participants who were considered clients of their organization. Participants were recruited between December 1, 2023, and January 31, 2024. All individuals who completed the 20-min survey and provided an email address received a $35 electronic Amazon gift card.

To ensure data quality and reduction of bot activity, our survey used Qualtrics survey protection, and the research team verified that the respondents’ IP addresses were in the United States. Additionally, data integrity was maintained by ensuring that each respondent could only complete the eligibility or survey questions once. Furthermore, a speeding check was used (respondents with a survey duration of ≤ one-third of the median duration of the survey), and participants who failed this quality check were excluded from the final sample. Qualtrics survey protection allowed us to use tools to prevent ballot box stuffing via a tool that places a cookie in the browser once a person has submitted a response, reCAPTCHA (Completely Automated Public Turing Test to tell Computers and Humans Apart) scores collected via a question placed before the survey asking respondents to identify specific items in pictures or replicate a series of letters, and bot detection via a Qualtrics survey question that indicates a reCAPTCHA score that relates to the probability that the respondent is a bot.

Upon clicking on the survey link, participants were provided with informed consent and assent (youth under 18) prior to participating in the study, ensuring they understood the purpose, procedures, and potential risks involved. They were then asked to complete a screening tool to assess their eligibility for the study. Individuals who met the inclusion criteria were asked a series of questions on demographics, developmental assets, such as academic engagement, and suicidal behaviors. Participants who used social media sites to complete the survey used personal computers or smart phones. Individuals who completed the survey in a community-based organization used a computer or tablet provided by the organization. The study was approved by The Ohio State University Institutional Review Board.

The inclusion/exclusion criteria were the same for all sampling sites. Respondents were eligible to participate in the study if they self-identified as Black or African American, were ages 14–24, resided in one of three Midwestern cities (Columbus, Detroit, and St. Louis), were assigned as male at birth, were fluent in English, currently identified as a male, and reported sexual contact (oral, anal, or otherwise) with a male in the previous year. Upon a disqualifying response, respondents who did not meet the inclusion criteria were immediately exited from the survey. We used the Qualtrics forced response option to ensure that every participant answered each question.

### Measures

#### Outcome variable: suicidal behaviors

The Columbia-Suicide Severity Rating Scale (C-SSRS) [21] is a clinician-administered interview used to assess both lifetime and recent suicidal ideation and behaviors. It evaluates suicidal thoughts related to death or suicide, as well as behaviors, including suicide attempts and non-suicidal self-injury. The "Suicidal Behaviors" section specifically focuses on the presence or absence of suicide attempts that occurred within the past three months and throughout the individual's lifetime. This includes actual attempts, interrupted attempts, aborted attempts, and preparatory behaviors. The assessment comprises four dichotomous items (answered as yes or no) related to suicidal behaviors. We calculated a total score for suicidal behaviors by summing the responses to these items. Additionally, if a participant reported a recent or lifetime suicide attempt, the number of attempts was documented as a continuous variable. The C-SSRS showed strong internal consistency in this study, with a Cronbach’s alpha of 0.87.

#### Independent variable: internal assets

*Positive identity* was measured using two subscales: hope and self-esteem. The hope subscale includes elements of personal power, sense of purpose, and a positive outlook on the future. Participants responded to items such as “I feel I do not have much to be proud of.” The Cronbach's alpha for the positive identity scale is 0.71. *Positive values* were assessed through four subscales: caring, social justice, integrity, and responsibility, with items like “Helping to make sure that all people are treated fairly.” The Cronbach's alpha for this scale is 0.89. *Social competencies* consisted of two subscales: social-emotional skills and planning/decision-making skills, with items like “Being good at making and keeping friends.” The Cronbach's alpha for this scale is 0.88. *Academic engagement* was measured with five items, including questions like “It bothers me when I don’t do something well.” The Cronbach's alpha for academic engagement is 0.72. All scales used a 5-point Likert-type scale ranging from 1 (Strongly disagree) to 5 (Strongly agree) [10].

### Demographic variables

The demographic variables in this study included household income, age, school enrollment, employment status, and highest education completed. Household income and age were treated as continuous variables, while the other variables were categorical. Specifically, school enrollment was coded as 0 (not enrolled) or 1 (enrolled); employment status was categorized as 1 (full-time), 2 (part-time), 3 (not employed), 4 (other), and 5 (student); and highest education completed ranged from 1 (never attended high school) to 12 (college degree, postgraduate, or professional school).

### Statistical analysis

Table 1 presents descriptive statistics summarizing participant characteristics. Table 2 displays the means and standard deviations of the measured internal assets. To explore relationships among the primary variables, Table 3 presents bivariate correlations highlighting interconnections between key study variables. The core analysis involves a multiple regression analysis, aimed at investigating the relationships between the covariates (age, household income, employment status, school enrollment, and education level), and independent variables, (social competencies, positive values, positive identity, and academic engagement) on the outcome variable suicidal behaviors. The findings from this analysis are detailed in Table 4. Notably, the study indicates that there is less than one percent missing data for this study. All statistical analyses were conducted using STATA version 18, ensuring rigorous and accurate computations throughout the study.

**Table 1:** Demographic variables among young BMSM ages 14–24 (*N* = 548)

Variable	Frequency	Percent
Age: *M* = 17; *SD* = 2.85; range = 14–24		
Household income: *M:* $75,000 to $99,999 annually; *SD* = *1.12*		
*Sexual orientation*		
Gay	464	84.6
Bisexual	70	12.7
Queer	10	0.2
Asexual	1	0.01
Pansexual	3	0.1
*School Enrollment*		
Yes	433	79
No	115	21
*Education Level*		
Never attended high school	4	0.4
Eighth grade	13	3
Ninth grade	26	5
Tenth grade	26	5
Eleventh grade	60	11
Twelfth grade	287	52
High school diploma or GED	79	14
Some college, associate’s degree, or trade school	40	7
College degree, postgraduate, or professional degree	11	2
*Employment Status*		
Full Time	93	17.29
Part Time	53	9.85
Not Employed	23	4.28
Other	11	2.04
Student	358	66.54
*Preparatory acts or behavior*		
No	325	60
Yes	213	40
*Aborted attempts*		
No	326	61
Yes	212	39
*Interrupted attempts*		
No	360	67
Yes	178	33
*Nonfatal suicide attempt*		
No	310	58
Yes	228	42

**Table 2:** Means and standard deviations of internal assets among young BMSM ages 14–24 (*N* = 538)

Internal assets	Mean	Standard deviation	Range
Positive identity	3.32	0.75	1–5
Positive values	4.00	0.82	1–5
Social competencies	4.00	0.80	1–5
Academic engagement	3.59	0.65	1–5

**Table 3:** Correlations of key study variables (*N* = 538)

Suicidal behaviors	1								
Positive values	− 0.25***	1							
Positive identity	− 0.34***	0.10*	1						
Social competencies	− 0.36***	0.75***	0.20***	1					
Academic engagement	− 0.16**	0.61***	0.21***	0.54***	1				
School enrollment	0.02	0.18***	0.03	0.08	0.18***	1			
Education level	− 0.00	− 0.01	− 0.07	0.04	− 0.013	− 0.08	1		
Employment status	0.03	0.10	0.10	0.07	0.02	0.63***	− 0.19***	1	
Age	− 0.18***	− 0.07	0.11	0.07	− 0.01	− 0.49***	0.33***	− 0.65***	1
Household income	− 0.03	0.09	0.06	0.07	0.02	0.37***	0.17***	0.43***	− 0.42***

^*^p < 0.05; ^**^p < 0.01, ^***^p < 0.001

**Table 4:** Multiple regression analysis on suicidal behaviors among youth (*N* = 538)

Variable	Unstandardized beta	Betas	Standard error	e
Positive values	− 0.59	− 0.24***	0.16	0.001
Positive identity	− 0.75	− 0.29^***^	0.11	0.001
Social competencies	− 0.70	− 0.29***	0.15	0.001
Academic engagement	0.48	0.16^**^	0.15	0.002
School enrollment				
Yes (reference)	− 0.37	− 0.07	0.27	0.173
*Education level*				
Never attended high school (reference)				
Eighth grade	− 1.65	− 0.12	1.47	0.263
Ninth grade	− 1.80	− 0.19	1.42	0.206
Tenth grade	− 1.13	− 0.11	1.42	0.429
Eleventh grade	− 1.70	− 0.26	1.40	0.225
Twelfth grade	− 1.08	− 0.27	1.38	0.432
High school diploma or GED	− 1.05	− 0.18	1.38	0.447
Some college, associate’s degree, or trade school	− 1.87	− 0.24	1.40	0.183
College degree, postgraduate, or professional degree	− 2.36	− 0.17	1.47	0.109
Household income	− 0.18	− 0.10*	0.04	0.029
Age	− 0.13	− 0.19**	0.03	0.003
*Employment status*				
Full Time (reference)				
Part-time	0.76	0.12*	0.34	0.026
Self-Employed	1.67	0.17***	0.46	0.001
Other	1.14	0.07	0.65	0.081
Student	0.36	0.08	0.32	0.254

^*^p < 0.05, ^**^p < 0.01, ^***^p < 0.001

## Results

The average age of the sample was 17 (*SD* = 2.85) and 85% self-identified as gay. The average household income was between $75,000 to $99,999 and 79% reported being enrolled in school. Thirty-three percent of the sample reported interrupted attempt at suicide and 42% reported non-fatal attempt at suicide (see Table 1). Young Black SMM reported high levels of positive values (*M* = 4.00; *SD* = 0.82). Young men also reported high levels of social competencies (*M* = 4.00; *SD* = 0.80). Lastly, for positive identity, Black SMM had a moderate mean score of 3.32 (*SD* = 0.75) (see Table 2).

### Bivariate regression analysis

The bivariate correlations for key study variables are listed in Table 3. For young Black SMM, positive values were associated with decreased suicidal behaviors (*r* = − 0.25; *p* < 0.001). Positive identity also was correlated with fewer suicidal behaviors (*r* = − 0.34; *p* < 0.001) and higher positive values (*r* = 0.09; *p* < 0.001). Social competencies (*r* = − 0.36; *p* < 0.001) was associated with fewer suicidal behaviors. Academic engagement was negatively correlated with suicidal behaviors (*r* = − 0.16; *p* = 002). School enrollment was associated with an increase in positive values (*r* = − 0.18; *p* < 0.001). Employment status was correlated with education level (*r* = 0.63; *p* < 0.001). Age was also correlated with fewer suicidal behaviors. (*r* = − 0.18; *p* < 0.001). Lastly, household income was negatively correlated with age (*r* = − 0.42; *p* < 0.001).

### Multiple linear regression analysis

Table 4 displays the results for the main regression models predicting suicidal behaviors. In addition, the model accounted for about 30% of the variance associated with suicidal behaviors. The findings indicated that positive identity was associated with lower levels of suicidal behaviors (β = − 0.29; *p* < 0.01). Positive values (β = − 0.24; *p* < 0.001) and social competencies (β = − 0.29; *p* < 0.01) also were associated with fewer suicidal behaviors. Academic engagement was associated with higher levels of suicidal behaviors (β = 0.16; *p* = 0.02). Household income was associated with lower suicidal behaviors (β = − 0.10; *p* = 0.029). Lastly, younger Black SMM were less likely to engage in suicidal behaviors in comparison to older Black SMM (β = -0.19; *p* = 0.03). Black SMM who reported working part-time (β = 0.15; *p* = 0.004), and self-employed (β = 0.20; *p* < 0.001), were all positively associated with suicidal behaviors in comparison to those who work full time.

## Discussion

LGBTQ youth report higher rates of suicidal behavior than their heterosexual counterparts [22]. Additionally, holding multiple marginalized identities, such as being a member of a racial minority group, is associated with elevated suicide risk and suicidal behavior [23–25].In this sample, 39% of participants reported having aborted a suicide attempt, 32% indicated that they had interrupted a suicide attempt, and 41% reported a nonfatal suicide attempt. These findings align with more recent studies of Black SMM aged 18–29, as well as other online samples of Black men aged 18–24 regarding suicidal behaviors [17, 18, 20, 26]. However, our results are notably higher than the figures reported by the Healthy Minds Study, which found that 14% of emerging adult students experienced suicidal ideation, 5.9% had aplan to diebysuicide, and 1.5% reported actual suicide attempts [27]. These disparities underscore the need for research focused on key strength-based approaches and interventions that leverage developmental assets to effectively support the mental health and well-being of young Black sexual minority males. 

Though the DAF aims to be comprehensive, little research exists on the application of DAF for young Black SMM. Further, to the best of our knowledge, this paper is the first to explore the application of internal assets for young Black SMM ages 14–24, specifically examining how internal assets such as positive identity, social competencies, personal values, and academic engagement are associated with suicidal behaviors.

The study’s findings suggest that positive values, positive identity, and social competencies are associated with reduced suicidal behaviors aligns with the limited available data on internal assets in this population. One study identified positive self-talk and self-encouragement, among 24 coping strategies identified among a racially diverse group of LGBTQ adolescents, and examined how these strategies contributed to improved positive identity [27]. Another study found that early development of a positive image of one’s sexual identity improved Black SMM well-being [28]. Additionally, a couple of studies found that religious values contributed to participants’ positive identitybyreinforcing their confidence and self-esteem [27, 28].

These findings underscore the critical need to cultivate internal assets that promote resilience among young Black SMM. By prioritizing strengths and nurturing positive identities, and other assets, we can create interventions tailored to effectively support this vulnerable population. This strength-based approach not only reduces the risk of suicidal behaviors but also enhances overall mental well-being. Furthermore, recognizing and amplifying these assets is essential for developing culturally relevant mental health strategies. Empowering young Black SMM to thrive requires an innovative framework that integrates their unique experiences and perspectives. Ultimately, fostering these internal strengths can lead to a brighter future for individuals and their communities.

Surprisingly, our results indicated that an increase in academic engagement is associated with higher levels of suicidal behaviors, which differs from our initial hypothesis. Thus, this study finding differs from numerous studies suggesting that external assets, such as positive, affirming, and supportive school environments build resilience and may be protective factor against Black SMM suicide.^29^ For Black sexual minority males, who often navigate societal pressures related to their identity, the stress associated with academic performance can lead to feelings of inadequacy and depression [29]. This, in turn, may heighten the risk of suicidal behaviors. Additionally, while increased academic engagement can foster personal development, it may not always facilitate social connections. Many Black SMMs may feel isolated in academic environments where they perceive themselves as different from their peers or experience discrimination as a result. This isolation can intensify feelings of loneliness and hopelessness, contributing to suicidal thoughts [30]. Furthermore, Black SMMs may struggle with inadequate coping mechanisms to manage the stressors linked to both academic demands and their minority identity. Without effective strategies to cope with these pressures, they may resort to maladaptive behaviors, further increasing their vulnerability to suicidal ideation [31, 32]. Creating inclusive environments that affirm the identities of Black SMM is essential in reducing feelings of alienation and enhancing their well-being. Schools must prioritize diversity training for both staff and students while implementing programs that celebrate and support LGBTQ + identities. Regular assessments of student well-being and engagement are critical. By analyzing data on mental health outcomes in relation to academic engagement, schools can inform targeted interventions and policies that promote healthier academic environments. Although academic engagement is crucial for youth development; it is vital to adopt a holistic approach that prioritizes mental health and emotional well-being. By establishing robust support systems and fostering inclusive educational environments, we can significantly reduce the risk of suicidal behaviors among Black sexual minority males, ultimately improving their overall academic experiences.

As this population holds multiple marginalized identities, it is also important to consider how the intersection of race compounds Black SMM mental health and well-being. Disparities continue to exist among Black individuals compared to White individuals seeking mental health care, and many of the underlying causes are attributed to structural racism [3]. Thus, positive identity impacts mental health care and access and likely indirectly impacts Black SMM mental health and well-being. While additional research is necessary to comprehensively elucidate this relationship, existing literature suggests that racial-ethnic socialization—the process through which individuals receive and internalize societal messages regarding their race, culture, and ethnic values—significantly influences their experiences and perceptions within the broader sociocultural context [18], can positively impact mental health and well-being [20]. Racial-ethnic socialization is often considered an external asset as it is dependent on how an individual’s family or community shapes that individual’s perspective of their race and culture, and there may be a potential relationship between individuals possessing a positive racial-ethnic socialization and those with positive identity [33]. More research is needed to understand how these two assets are connected.

### Limitations

While the current study enhances our understanding of developmental assets and their positive impact on Black SMM suicidal behaviors, it is important to acknowledge its limitations. One key limitation is that not all 40 developmental assets were utilized, precluding the ability to identify additional assets that might lower suicidal behaviors among Black SMM. Furthermore, the study sample consisted of young Black SMM in the Midwest, which may not be representative of the broader population. This limitation could affect the generalizability of the findings. Moreover, the cross-sectional research design does not allow for the establishment of causality. The simultaneous examination of both the outcome and the exposure variables prevents the determination of a temporal relationship between them. Future research efforts could address these limitations to provide more robust and nuanced insights into the factors influencing suicidal behaviors among young Black SMM.

### Practice implications

To effectively address the health disparities experienced by young Black sexual minority males, practitioners, must adopt an intersectional approach that recognizes the complex interplay of race, sexual orientation, and social determinants of health. This includes acknowledging the structural and relational power dynamics—such as institutional racism, anti-Blackness, and homonegativity—that shape and sustain these disparities, which the intersectionality theoretical framework explicitly interrogates. Given that only about one in three Black LGBTQ youth have access to supportive external resources, it is imperative for professionals in the field to identify and build upon the internal assets inherent in this population [17, 18, 20]. Practitioners should focus on enhancing the internal strengths and resilience of young Black SMM through targeted interventions. This may involve creating safe and affirming environments where clients can explore their identities and experiences, as well as developing programs that foster self-advocacy and community engagement. By prioritizing the strengths of Black SMM, practitioners can empower them to navigate the unique challenges they face, thereby promoting better mental health and overall well-being. Moreover, social workers and other practitioners should integrate an understanding of the broader sociocultural context in which their clients operate. This includes being aware of systemic barriers and societal stigmas that may impact the lives of young Black SMM, while also recognizing the cultural resources that exist within their communities. Collaboration with community organizations and stakeholders is essential to enhance social support networks and resources for young Black SMM. By working together, practitioners can create comprehensive support systems that address both internal strengths and external challenges. Ultimately, a holistic approach that incorporates the internal assets of Black SMM, alongside awareness of the broader sociocultural factors at play, can lead to more effective public health initiatives and improved health outcomes for young Black sexual minority males.

### Public health implications

Public health concerns surrounding mental health and well-being have garnered significant attention, especially within marginalized communities. For Black SMM aged 14–24, understanding how internal assets—such as positive identity, positive values, and academic engagement—are cultivated is vital in addressing suicidal behaviors. Moreover, supporting young Black SMM necessitates a comprehensive approach centered on enhancing these internal assets. By fostering supportive environments and promoting positive identity, academic involvement, and constructive values, public health initiatives can play an essential role in mitigating suicidal behaviors within this vulnerable demographic. It is crucial that we advocate for continuous resources, education, and open dialogues that empower these young men to overcome adversity and flourish.

## Conclusion

Black SMM experience greater rates of suicide than their peers, including other Black youth. Internal developmental assets such as mattering and belonging, social connectedness, and positive identity may be important factors in reducing suicide planning, attempts, and completions; however more research is needed to understand the mechanisms by which internal assets impact Black SMM mental health and well-being and how these assets can be integrated into spaces Black SMM frequent.

## Data Availability

Data that support the findings of this study are available on request under a license agreement. Written applications can be made to the author corresponding author (boyd.465@osu.edu).
